# The deficiency of *htr6* gene results in aberrant brain development and epileptiform discharges in zebrafish larvae

**DOI:** 10.7150/ijms.135746

**Published:** 2026-10-02

**Authors:** Hongyuan Chu, Hao Wang, Sijia Wen, Renqiuguo Li, Junjiao Zhang, Fan Zhang, Huaxia Luo, Ye Wu, Jingmin Wang, Kai Gao, Yuwu Jiang

**Affiliations:** 1 Children's Medical Center, Peking University First Hospital, Beijing, China.; 2 Beijing Key Laboratory of Molecular Diagnosis and Study on Pediatric Genetic Diseases, Beijing, China.; 3 Children Epilepsy Center, Peking University First Hospital, Beijing, China.; 4 Key Laboratory for Neuroscience, Ministry of Education/National Health and Family Planning Commission, Peking University, Beijing, China.; 5 Center of Epilepsy, Beijing Institute for Brain Disorders, Beijing, China.

**Keywords:** zebrafish, neurodevelopment, epilepsy, transcriptome analysis

## Abstract

**Background:**

The 5-Hydroxytryptamine Receptor 6 (HTR6) is a G protein-coupled receptor implicated in neurodevelopmental disorders and epilepsy. Our previous studies identified a somatic *HTR6* mutation in brain surgical specimens from patients with focal cortical dysplasia type II, suggesting its potential involvement in disease pathogenesis.

**Objective:**

To investigate the functional role of the *htr6* in zebrafish, focusing on its impact on neurodevelopment and seizure susceptibility.

**Methods:**

A F0 mosaic *htr6* crispant zebrafish model was generated using CRISPR/Cas9 and validated by sequencing and TIDE analysis. Morphological and central nervous system (CNS) abnormalities were assessed at 5 days post-fertilization (dpf) by bright-field and Tg (HuC: eGFP) fluorescence imaging. Survival was monitored to 21 dpf. Locomotor behavior was evaluated using DanioVision, and epileptiform activity was examined by local field potential recording. Human *HTR6* mRNA rescue experiments, transcriptome sequencing, and qPCR validation were also performed.

**Results:**

Deficiency of *htr6* resulted in significant reductions in interocular and central nervous system areas. In addition, F0 mosaic *htr6* crispant showed significantly reduced survival compared with Cas9 controls. While locomotor activity was markedly unaffected, electrophysiological recordings revealed epileptiform events in 9 of 24 F0 mosaic *htr6* crispant but none in the Cas9 controls. Co-injection of human *HTR6* mRNA rescued both neurodevelopmental abnormalities and epileptiform activity. Transcriptome analysis identified 402 differentially expressed genes, including the upregulation of 355 and the downregulation of 47 genes, suggesting profound neuronal impacts from *htr6* deficiency. KEGG pathway enrichment analysis linked these changes to ABC transporters and metabolic pathways, demonstrating the significance of *htr6* in maintaining neuronal health.

**Conclusion:**

Deficiency of *htr6* in zebrafish results in abnormal brain development and epileptiform activity, underscoring its critical role in neurodevelopment and neuronal excitability. These findings suggest the importance of *HTR6* in neurodevelopment and epilepsy, providing a foundation for further research on therapeutic strategies targeting *HTR6*-related pathways in neurological disorders.

## Introduction

Serotonin, also referred to as 5-hydroxytryptamine (5-HT), exerts a range of physiological and pharmacological responses by interacting with various receptor subtypes^1^, ^2^. 5-HT receptor 6 (HTR6) is a relatively novel addition to the serotonin receptor family^3^. Sharing less than 40% of homological sequence^4^, it differs in both structural and pharmacological features from any other known G-protein coupled 5-HT receptors. It is exclusively expressed in the central nervous system (CNS)^5^. Temporal studies show dynamic changes of HTR6 localization, from the soma of stem cells and neuronal somatodendritic compartments during embryonic development to mainly the hippocampus, cerebral cortex, and striatum in adulthood^5^, regions that are associated with higher cognitive functions^6^. However, the physiological role of HTR6 during brain development and epileptogenesis remains poorly understood.

Recent studies have underscored that HTR6 plays an important role in modulating seizure activity. Selective HTR6 antagonists could increase seizure threshold in rats, making them potential therapeutic target for epilepsy^3^, ^7^. Our previous study identified a potentially deleterious somatic variant in *HTR6* (c.G469A, p. Ala157Thr, NM_000871.3) in postsurgical brain tissue from 1 out of 17 children with focal cortical dysplasia (FCD) type IIb^8^. However, gaps persist in our understanding of the precise mechanisms by which HTR6 influences neurodevelopment and seizure activity. This indicates a pressing need for further investigation into the role of HTR6 in the context of epilepsy and related neurodevelopmental disorders.

The zebrafish genome has a *HTR6* orthologue (*htr6*, ENSDARG00000016095). Comparative alignment of human *HTR6* and zebrafish *htr6* shows substantial conservation, particularly within the 7-transmembrane helix (Figure S1 and Table S1). Given this evidence, zebrafish larvae were employed to investigate the causal relationship between HTR6 and neurodevelopment. In this study, we investigated the phenotypic consequences of *htr6* deficiency in zebrafish using CRISPR/Cas9-mediated gene editing techniques, which is an established model organism in neurobiological research. Finally, to further elucidate the molecular pathways influenced by *htr6* deficiency in zebrafish, we have conducted transcriptome sequencing. These results reveal the role of HTR6 in neurodevelopment and epilepsy.

## Methods

### Zebrafish maintenance and breeding

Tübingen zebrafish strain was reared in recirculating water under standard laboratory conditions^9^. The tank temperature was generally between 28-29 ℃, while the dark-light cycle was set to 10:14, and fish were fed twice daily. Embryos utilized for the study were obtained from in-tank breeding, during which adults had a male-to-female ratio of 1:1. Then the embryos were transferred to a 100mm Petri dish with E3 embryo medium (composed of 0.03% Instant Ocean and 0.0002% methylene blue in reverse osmosis-distilled water), with a maximum of 60 larvae per dish. Larvae were not fed before 5 days post-fertilization (dpf) in accordance with established practice, and from 5 dpf onward, larvae were fed twice daily with commercial juvenile fish diet, and 20% of the medium was replenished with fresh E3 solution.

### Cas9 control and *htr6* deficiency zebrafish

Zebrafish embryos derived from the same clutch and the same parental pair were randomly selected for microinjection. Following injection with either Cas9 protein alone or Cas9 protein combined with *htr6*-targeting small guide RNA (sgRNAs), embryos were allocated to separate Petri dishes containing E3 medium and maintained in an incubator until the day of experimentation. The zebrafish model with deficiency of *htr6* was obtained by injecting embryos with sgRNA and Cas9 protein at the one-cell stage, generating F0 mosaic crispants rather than stable germline mutants.

The sgRNAs were designed to target the first exon of zebrafish *htr6*, far upstream from the 50 amino-acid residues before the coding sequence stop codon. After microinjecting Cas9 protein combined with individual sgRNAs into zebrafish embryos, Tracking of Indels by Decomposition (TIDE, https://tide.nki.nl/) algorithm quantification showed sgRNA1 and sgRNA4 achieved average editing efficiencies of 82% and 71% respectively, far exceeding sgRNA2 (12%) and sgRNA3 (16%). Collectively, sgRNA1 and sgRNA4 are selected as high-efficiency guides for subsequent experiments. Polymerase chain reaction (PCR) and Sanger sequencing were performed using forward primer CTACCTCCTCATTATCTCCCCG and reverse primer GTGGCTCCTGATGACTGTAGT to validate. Editing efficiency was assessed using TIDE. Post hoc genotyping was performed after the phenotypic studies. Individual larvae were randomly collected for sequencing and TIDE analysis to confirm the mutagenesis. Individual data about gene editing efficiency are provided in Table S2 and Table S3.

### Microscope observation

Normal zebrafish larvae and Tg (HuC: eGFP) larvae at five dpf were placed into a well-plate with dorsal side up for bright-field and fluorescent imaging separately. Images were acquired with Nikon SMZ800N microscope and Touptek high-resolution CCD camera. Measured traits were: Eye distance, body length, eye/body ratio, interocular area, and CNS subregions, including the forebrain, midbrain, and hindbrain. Interocular distance is a widely used morphological proxy for brain morphogenesis in zebrafish larvae, reflecting the coupled development of the eye field and forebrain^10^. Area measurements in photos were performed using ImageJ.

### Survival analysis

Embryos were allocated to either a Cas9 control group and F0 mosaic *htr6* crispant group experimental group and maintained in E3 medium at 28-29 °C. Unfertilized eggs and embryos displaying physical damage from microinjection were removed at 1 dpf. At 9:00 and 17:00 each day, deceased individuals were removed. Survival was recorded daily until 21 dpf, at which point final survival rates were analyzed, and survival curves were generated.

### Locomotor behavior assay

Zebrafish larvae at 5 dpf were tracked for locomotor behavior activity with DanioVision (Noldus). 15-minutes tracks after 30 minutes of habituation were recorded for spontaneous behavior, afterwards, further locomotor behavior at 5-min dark/light epochs was also taken down. For each larva, the mean movement distance per 30 seconds and the corresponding standard error, as well as the mean maximum acceleration and the corresponding standard error, were calculated and plotted as line graphs. For spontaneous activity, the total movement distance and maximal acceleration during the 15-min recording were quantified for statistical comparison. Because behavioral responses to the dark/light epochs were not significant during the first cycle, data from that cycle were excluded from analysis. Data from the second to fourth cycles were averaged at corresponding time points and used for statistical evaluation.

### Electrophysiological recording

Electrophysiological recording was performed in zebrafish larvae at 5 dpf. Larvae from the Cas9 control group and F0 mosaic *htr6* crispant group were paralyzed in 50 µM pancuronium (Sigma) and immobilized in 2% low-melting point agarose within a recording chamber. Local field potentials were recorded by microelectrode implanted through optic tectum, amplified through A-M system, band-pass filtered online at 1 Hz - 5 kHz, digitized at a sampling rate of 10 kHz using a Digidata digitizer, and acquired with DClamp software. Each electrophysiological recording was conducted for 15 minutes per zebrafish larva. Epileptiform events were identified and categorized according to previously established criteria^11^. Ictal-like events were defined as multi-spike discharges exceeding 5× baseline noise and lasting more than 500 ms. Interictal-like events were defined as discharges with amplitudes exceeding 3× baseline noise and durations greater than 100 ms. The occurrence of electrographic epileptiform activity, including both ictal- and interictal-like events, was quantified for analysis.

### Human *HTR6* rescue experiment

The human *HTR6* wild type cDNA was subcloned into vector pcDNA3.1, yielding the pcDNA3.1-*HTR6*-wt plasmid. The specific *HTR6* cDNA insert was gel-purified and transcribed using the T7 High Yield RNA Transcription kit (No. TR101-01, Vazyme, China) following the manufacturer's protocol. This RNA then underwent capping with the Vaccinia Capping System kit (No. TR101-01, Yeasen, China), followed by poly(A) tailing using the E. coli Poly(A) Polymerase kit (No. DD4111-01, Vazyme, China). Finally, the RNA was purified with the RNA Purification kit (No. DP42, Tiangen, China) to produce *HTR6*-wt mRNA. In vitro-synthesized *HTR6*-wt mRNA was combined with sgRNA and Cas9 protein, then injected into 1- to 2-cell stage zebrafish embryos to generate the human *HTR6* rescue model.

### Transcriptome sequencing

Transcriptomic analyses were conducted on an independent batch of zebrafish larvae, prepared and treated under identical experimental conditions. Four biological replicates per group (Cas9 control and F0 mosaic *htr6* crispant) were analyzed. Each replicate consisted of pooled tissues from approximately 20 larval heads. The spectrophotometer was utilized to evaluate the concentration and the purity of RNA samples. 2100 RNA nano 6000 assay kit and Agilent 2100 Bioanalyzer were utilized to evaluate the integrity of RNA samples. After quality control, TIANSeq mRNA Capture Kit was utilized to capture RNA with poly-A. Illumina kit was utilized to construct the library. Concentration was quantified and then diluted, followed by insert-size check and quantitative PCR. After clustering, the library was sequenced using the Illumina platform.

Reads of about 150 base pairs were generated and stored in fastq format. Q20, Q30, and GC content were calculated and reads containing adapters or with low quality were trimmed. Paired-end read alignment to the reference genome was performed utilizing Hisat2. Fragments Per Kilobase of transcript per Million mapped reads (FPKM) of each gene was calculated based on read counts and length-of-gene. Differential expression analysis was performed using DESeq2 under R 4.4.1. To control false discovery rate, *p*-values were adjusted using the Benjamini and Hochberg's approach. Genes with adjusted *p* < 0.05 were regarded as differentially expressed. Gene enrichment analysis of differentially expressed genes was performed utilizing topGO and clusterProfiler in R. Protein-protein interaction (PPI) analysis for the differentially expressed genes was performed according to the STRING database and visualized using cytoscape v3.10.3.

### RNA extraction and qPCR

Larvae were harvested for RNA extraction utilizing RNA-easy Isolation Reagent (No. R701-01, Vazyme, China). Forty larvae from each group—Cas9 control and F0 mosaic *htr6* crispant—were collected. Homogenization was performed with 10 larvae per tube, totaling four tubes per group. RNA concentrations were then quantified spectrophotometrically (NanoDrop). Subsequently, 1 μg of total RNA from each sample underwent reverse transcription using the HiScript III 1st Strand cDNA Synthesis Kit (No. R312-01, Vazyme, China), strictly adhering to the manufacturer's protocol. Quantitative real-time PCR (qPCR) analysis was conducted employing AceQ qPCR SYBR Green Master Mix (No. R111-02, Vazyme, China). The *actb* gene, encoding β-actin, served as the internal control. Primer sequences utilized for qPCR are detailed in Table S4. Equipment and reagent utilized in the current study are shown in Table S5.

### Statistical analysis

Sequencing quality was assessed, and samples that failed sequencing or exhibited insufficient editing efficiency (< 5%) were subsequently filtered from analysis. Detailed sample sizes and exclusion criteria for each assay are summarized in Table S6. Statistical analysis and plotting were conducted using GraphPad Prism 10. The Shapiro-Wilk test evaluated the normality of continuous variables. For data conforming to a normal distribution with equal variances, unpaired two-tailed Student's *t*-tests compared two groups, with error bars representing the standard deviation (SD). For non-normal data, the Mann-Whitney U test was applied, and error bars depict the interquartile range (IQR). Additionally, the Kolmogorov-Smirnov test was applied to evaluate whether the two continuous variables follow the same distribution. Categorical variables were analyzed using Chi-squared tests or Fisher's exact test, as appropriate. Survival curves were assessed via the log-rank test. Interval-based death distribution analysis was further applied to quantitatively define stage-specific mortality windows by calculating the proportion of mortality events occurring within canonical developmental intervals (0-5 dpf, 6-15 dpf, 16-21 dpf). Multi-group comparisons of non-normal data employed the Kruskal-Wallis test, followed by Dunn's multiple comparisons test for post hoc analysis. Details regarding the statistical method for the Light-Dark behavior assay are provided in the Appendix. Specific statistical tests are indicated in each figure legend. A *p*-value < 0.05 was considered statistically significant for all tests, where * denotes *p* < 0.05, ** denotes *p* < 0.01, *** denotes *p* < 0.001, and **** denotes *p* < 0.0001.

## Results

### Deficiency of *htr6* impairs early brain morphological development in zebrafish larvae

As shown in **Figure 1A**, both sgRNAs were designed to target the first exon of *htr6*, with sgRNA1 positioned between transmembrane (TM) helices 4 and 5 and sgRNA4 near TM helices 5 and 6. Representative figures depicting zebrafish larvae from the Cas9 control (n = 20) and the F0 mosaic *htr6* crispant (n = 25) in light epochs were shown as **Figure 1B**. Eye distance and body length were marked in blue, and the interocular area was delineated with a dotted line. At 5 dpf, F0 mosaic *htr6* crispant exhibited normal eye distance [**Figure 1C-D**, Cas9: 0.480 ± 0.0178 mm, *htr6*: 0.472 ± 0.0208 mm, mean ± SD, Unpaired *t*-test, t(43) = 1.386, and *p-value* = 0.173], body length [**Figure 1E-F**, Cas9: 3.853 ± 0.113 mm, *htr6*: 3.789 ± 0.138 mm, mean ± SD, Unpaired *t*-test, t(43) = 1.684, and *p-value* = 0.099], and eye/body ratio [**Figure 1G-H**, Cas9: 0.125 ± 0.00, *htr6*: 0.125 ± 0.01, mean ± SD, Unpaired *t*-test, t(43) = 0.066, and *p-value* = 0.948]. However, a significant decrease in interocular area was found in F0 mosaic *htr6* crispant [**Figure 1I-J**, Cas9: 0.0774 ± 0.00515 mm^2^, *htr6*: 0.0719 ± 0.00651 mm^2^, mean ± SD, Unpaired *t*-test, t(43) = 3.074 and *p-value* = 0.004].

**Figure 2A** depicted representative imaging of HuC: eGFP expressed larval zebrafish, exhibiting a CNS fluorescence pattern at 5 dpf. A white dashed line highlights the CNS subregions, including the forebrain, midbrain, and hindbrain, for measurement. Compared with the Cas9 control (n = 25), there was a significant decrease in the whole CNS area in the F0 mosaic *htr6* crispant (n = 27) [**Figure 2B-C**, Cas9: 0.205 (0.196, 0.209) mm^2^, *htr6*: 0.184 (0.177, 0.195) mm^2^, Median (IQR), Mann-Whitney U test, *p-value* < 0.0001]. Similarly, the forebrain area [**Figure 2D-E**, Cas9: 0.0330 ± 0.00259 mm^2^, *htr6*: 0.0299 ± 0.00312 mm^2^, mean ± SD, Unpaired *t*-test, t(50) = 3.891 and *p-value* = 0.0003], midbrain area [**Figure 2F-G,** Cas9: 0.0819 (0.0759, 0.0848) mm^2^, *htr6*: 0.0728 (0.0710, 0.0769) mm^2^, Median (IQR), Mann-Whitney U test, *p-value* < 0.0001] and hindbrain area [**Figure 2H-I**, Cas9: 0.0876 ± 0.00501 mm^2^, *htr6*: 0.0821 ± 0.00409 mm^2^, mean ± SD, Unpaired *t*-test, t(50) = 4.327 and *p-value* < 0.0001] were significantly decreased in F0 mosaic *htr6* crispant. Although the magnitude of reduction varied among individual larvae, the majority of F0 mosaic *htr6* crispants displayed smaller measurements than controls. Individual morphological data for each zebrafish are provided in the Appendix for reference.

### Deficiency of *htr6* in zebrafish increases epileptiform signal

Electrophysiological recordings were analyzed between the Cas9 control group (n = 25) and the F0 mosaic *htr6* crispant group (n = 24) at 5-6 dpf. No epileptiform signals were detected in any of the 25 Cas9 controls. In contrast, in the initial characterization experiment, 9 out of 24 F0 mosaic *htr6* crispants were observed to present spontaneous epileptiform events. Among the affected F0 mosaic *htr6* crispant, the number of events per 15 minutes recording period ranged from 2 to 10 (mean±SD: 4.78±2.68 events/15 min), with a median of 4 events. The average duration of individual epileptiform events varied widely across zebrafish, from 534.5 ms to 1024.5 ms (mean±SD: 734.4±192.2 ms), and the cumulative seizure time per fish over the 15 minutes epoch ranged from 1652 ms to 5927 ms (mean±SD: 3279.4±1600.2 ms). Fisher's exact test confirmed a highly significant difference in the incidence of epileptiform signals between the two groups (*p-value* < 0.001). This is illustrated in **Figure 3, A-F**.

### Deficiency of *htr6* in zebrafish has no effect on locomotor behavior but reduces survival

Records of spontaneous behavior (15-minute tracks after 30 minutes of habituation, shown as **Figure 4A**) in Cas9 control (n = 39) and F0 mosaic *htr6* crispant (n = 32; one larva was excluded due to tracking loss) are presented in **Figure 4B-E**. No significant differences were observed in either displacement distance [**Figure 4B-C**, Cas9: 1589 ± 376.0 mm, htr6: 1489 ± 168.3 mm, mean ± SD, Unpaired *t*-test, t(69) = 1.382 and *p-value* = 0.172] or maximum acceleration [**Figure 4D-E**, Cas9: 769.2 ± 501.9 mm/s^2^, htr6: 749.4 ± 461.0 mm/s^2^, mean ± SD, Unpaired *t*-test, t(69) = 0.171 and *p-value* = 0.865]. For the subsequent 5-min dark/light epoch assay (**Figure 4F-I**), locomotor behavior was analyzed in Cas9 control (n = 39) and F0 mosaic *htr6* crispant (n = 33). No significant differences were found in both the light epoch [displacement distance: **Fig 4F**, Cas9: 877.0 ± 102.9 mm, *htr6*: 895.1 ± 92.3 mm, mean ± SD, Unpaired t-test, t(70) = 1.578 and *p-value* = 0.119; maximum acceleration: **Fig 4H**, Cas9: 1001 ± 303.3 mm/s^2^, *htr6*: 969.9 ± 375.4 mm/s^2^, mean ± SD, Unpaired *t*-test, t(70) = 0.567 and *p-value* = 0.573] or the dark epoch [displacement distance: **Fig 4F**, Cas9: 755.6 ± 133.6 mm, *htr6*: 807.8 ± 147.1 mm, mean ± SD, Unpaired *t*-test, t(70) = 0.781 and *p-value* = 0.437; maximum acceleration: **Fig 4H,** Cas9: 1183 ± 275.5 mm/s^2^, *htr6*: 1220 ± 272.2 mm/s^2^, mean ± SD, Unpaired *t*-test, t(70) = 0.389 and *p-value* = 0.699].

To specifically evaluate whether larvae exhibiting epileptiform signal displayed altered spontaneous locomotor behavior, we retrospectively stratified the animals according to the presence (n = 9) or absence (n = 15) of epileptiform signal identified from electrophysiological recordings, and compared these groups with the Cas9 control group (n = 39). No significant differences were detected in spontaneous locomotor parameters across the three groups. For total distance moved, statistical analysis showed (**Figure S2a**, Cas9: 1589 ± 376.0 mm, *htr6*-baseline: 1454 ± 145.3 mm, *htr6*-epileptiform: 1599 ± 208.6 mm, mean ± SD; Cas9 vs.* htr6*-baseline, *p-value* = 0.3498; Cas9 vs. *htr6*-epileptiform, *p-value* = 0.9955; *htr6*-baseline vs. *htr6*-epileptiform, *p-value* = 0.5271). Similarly, maximum acceleration did not differ significantly (**Figure S2b**, Cas9: 769.2 ± 501.9 mm/s^2^, *htr6*-baseline: 666.0 ± 541.4 mm/s^2^, *htr6*-epileptiform: 766.8 ± 247.9 mm/s^2^, mean ± SD; Cas9 vs. htr6-baseline, *p-value* = 0.7652; Cas9 vs. *htr6*-epileptiform, *p-value* > 0.9999; *htr6*-baseline vs. *htr6*-epileptiform, *p-value* = 0.8755). Tukey's multiple-comparisons test was used for all post-hoc assessments.

As illustrated in **Figure 4J**, a significant difference in survival rates was observed between the Cas9 control group (n = 50) and the F0 mosaic *htr6* crispant group (n = 56) (Log-rank test, *p-value* = 0.001). At 21 dpf, the survival rate was 64.00% in the Cas9 control group, whereas it was 35.71% in the F0 mosaic *htr6* crispant. Interval-based mortality distribution analysis showed that 87.5% of all *htr6*-deficient larval mortality occurred within the 6-15 dpf developmental window, whereas only 12.5% of deaths occurred before 5 dpf, and no mortality was observed after 15 dpf.

### Human *HTR6* rescues the CNS developmental abnormalities and epileptiform signal in F0 mosaic *htr6* crispant group

To test whether reduced *htr6* expression underlies the CNS abnormalities and epileptiform activity observed in F0 mosaic *htr6* crispant (shown as **Figure 5A**), we performed a human *HTR6* rescue. Embryos co-injected with human *HTR6* mRNA and *htr6* CRISPR reagents (*HTR6* rescue group) exhibited significantly larger whole-CNS area (**Figure 5B-C,** Cas9: 0.155 (0.146, 0.160) mm^2^, *htr6*: 0.142 (0.135, 0.148) mm^2^, *HTR6*-rescue: 0.154 (0.145, 0.162) mm^2^, Median (IQR); Kruskal-Wallis test, *p-value* = 0.0006), midbrain area (**Figure 5F-G**, Cas9: 0.0629 (0.0588, 0.0661) mm^2^, *htr6*: 0.0579 (0.0547, 0.0612) mm^2^, *HTR6*-rescue: 0.0636 (0.0547, 0.0673) mm^2^, Median (IQR); Kruskal-Wallis test, *p-value* = 0.012), and hindbrain area (**Figure 5H-I**, Cas9: 0.0677 (0.0651, 0.0729) mm^2^, *htr6*: 0.0635 (0.0599, 0.0674) mm^2^, *HTR6*-rescue: 0.0691 (0.0647, 0.0739) mm^2^, Median (IQR); Kruskal-Wallis test, *p-value* = 0.0007) compared with F0 mosaic *htr6* crispant, with no significant difference from Cas9 control. For the forebrain area (**Figure 5D-E**), although the pairwise comparison between htr6 and rescue groups did not reach statistical significance, a trend toward recovery was observed (Cas9: 0.0232 (0.0196, 0.0265) mm^2^, *htr6*: 0.0199 (0.0177, 0.0222) mm^2^, *HTR6*-rescue: 0.0220 (0.0202, 0.0243) mm^2^, Median (IQR); Kruskal-Wallis test, *p-value* = 0.011; Dunn's post-hoc *htr6* vs. *HTR6*-rescue, *p-value* = 0.093). Electrophysiologically (representative as **Figure 6A-C**), in the rescue experiment cohort, epileptiform activity occurred in 1/31 larvae in the *HTR6* rescue group versus 15/32 in F0 mosaic *htr6* crispants (**Figure 6D**; Fisher's exact test, *p* < 0.001) and did not differ between the *HTR6* rescue and Cas9 control groups (Fisher's exact test, *p* > 0.999). Together, these data indicate that restoring *HTR6* expression rescues both the neurodevelopmental and seizure-related phenotype.

### Transcriptome sequencing revealed dysregulated mTOR, ABC transporter, and synaptic signaling pathways

To identify differentially expressed genes (DEGs) after *htr6* deficiency, transcriptome sequencing technique was performed. mRNA from Cas9 control (n = 4) and F0 mosaic *htr6* crispant (n = 4) were collected and measured. Of the 402 DEGs, 47 were down-regulated and 355 were up-regulated (as shown in **Figure 7A**). The volcano profile of the up-regulated and down-regulated genes was shown in **Figure 7B**. Notably, mechanistic target of rapamycin (mTOR) -related genes *slc3a2b*,* ulk2*,* pik3r1*,* sgk1*,* ddit4*,* sgk2b*,* slc3a2a*,* akt2l*,* eif4bb*,* rnf152* were significantly up-regulated, while *fzd8a*,* fzd3a*,* fzd10*,* fzd5*,* fzd1*,* atp6v1b2*,* strada*,* eif4ba*, *prkcba* were down-regulated, which passed the false discovery rate adjustment. To determine related functions of DEGs after *htr6* deficiency, Gene Ontology (GO) enrichment was performed for all DEGs. The result is shown in **Figure 7C**. The ATP-binding cassette (ABC) -type transmembrane transporter activity and transmembrane transport were significantly enriched. Besides, we identified significant enrichment of synaptic-related terms, including synaptic vesicle cycling, neurotransmitter transport, postsynaptic density organization, and presynaptic active zone components. Specifically, many DEGs were annotated to synaptic processes such as vesicle exocytosis (*unc13b*), endocytosis (*ap3b2, epn1*), trans-synaptic signalling (*bdnf, lrrk2*), and structural constituents of the postsynaptic density (*actn4*, *dlg* family) and presynaptic membrane (*cacna2d3*,* flrt2*). Kyoto Encyclopedia of Genes and Genomes (KEGG) enrichment analysis was also performed. ABC transporters pathway (ko02010), and a series of metabolism pathways were mainly enriched, as the top pathways were shown in the dot plot as **Figure 7D**. Protein-protein interaction analysis of significant differentially expressed mTOR pathway genes, ABC superfamily genes, and neural and synaptic pathway genes were shown in **Figure 7E**. Besides, through qPCR analysis, we confirmed the upregulation of *akt2l, pik3r1*, and the downregulation of *fzd8a*, which were mTOR-related genes (Figure S3).

## Discussion

The current study investigated the role of the *htr6* gene in zebrafish neurodevelopment, utilizing genetic manipulation techniques. The results revealed that *htr6* deficiency triggered a notable reduction in CNS areas, accompanied by a substantial surge in epileptiform signals and diminished survival rates. Transcriptome sequencing further revealed altered gene expression profiles following *htr6* deficiency, with significant enrichment in mTOR-related pathways, along with the ABC transporters and various metabolism pathways, suggesting that *htr6* may contribute to epilepsy through the regulation of molecular mechanisms involved in neuropathology. The findings from this research not only fill existing knowledge gaps regarding *htr6*'s function but also provide insights into potential genetic targets for therapeutic interventions in epilepsy and neurodevelopmental disorders.

*HTR6* is a protein-coding gene located on chromosome 1p36.13. The 5-HT6 receptor is a canonical G-protein-coupled receptor, while accumulating studies have identified its key regulatory function in neural development^12^. MAO-A knockdown during embryogenesis causes structural brain abnormalities linked to reduced apoptotic activity and elevated serotonin levels. Excessive 5-HT activates 5-Htr6 receptor, inhibiting caspase-3 and -9, enhancing antiapoptotic proteins, and leading to developmental retardation in the brain^13^. Dysfunction of the serotonergic system is linked to suicide, with genetic studies highlighting the role of serotonergic genes. The 5-HT6 receptor gene 267C/T SNP was examined in a Portuguese population, revealing a non-significant association with suicide overall, but a significant gender-specific association in male subjects^14^. A genome-wide association study performed in over 40,000 patients with bipolar disorder and 371,549 controls established *HTR6* as an expression quantitative trait locus for bipolar disorder^15^, which further supports the involvement of *HTR6* in the pathogenesis of multiple psychiatric and neurologic disorders^5^, ^12^, ^16^. Thus, the pleiotropy and wide phenotype spectrum of *HTR6* might explain both epilepsy and psychiatric comorbidities. In our previous study, we identified a possible deleterious somatic variant in *HTR6* on resected brain lesional tissues of a patient with FCD IIb^8^. These investigations led to the identification of the *HTR6* variant as a potentially new candidate pathogenic variant for FCD.

Our findings revealed that deficiency of *htr6* resulted in significant reductions in interocular area and CNS area, highlighting its critical role in early brain morphogenesis. The decreased brain regional volume may reflect impaired neuronal proliferation and differentiation, leading to abnormal cortical layering and excitatory-inhibitory imbalance, key contributors to FCD and epileptogenesis^17^. Importantly, the structural alterations observed in the F0 mosaic *htr6* crispant model suggest potential disruptions in serotonergic signaling pathways, which have been implicated in modulating neuronal excitability and seizure thresholds^13^, ^18^. Such changes could predispose to hyperexcitability and abnormal network synchronization, consistent with the clinical hallmark of FCD. Consistent with this interpretation, electrophysiological recordings revealed spontaneous epileptiform discharges in a subset of F0 mosaic *htr6* crispant, offering concrete functional evidence of inherent network hyperexcitability even without external provocation. The emergence of spontaneous epileptiform activity bolsters the hypothesis that *HTR6* deficiency lowers seizure threshold and fosters pathological synchronization, thereby forging a direct link between early neurodevelopmental defects and subsequent epileptogenic network states. Together, these morphological and electrophysiological findings strengthen the mechanistic connection between HTR6 dysfunction and seizure generation. Overall, by linking the morphological findings with functional outcomes, our study provides novel insights into how serotonergic receptor abnormalities can drive both developmental and epileptogenic consequences. These results not only enhance our understanding of seizure-related pathophysiology in FCD but also suggest that targeting HTR6 signaling may be a potential therapeutic avenue.

Although spontaneous epileptiform activity emerged in a subset of *htr6*-deficient larvae, our locomotor assays revealed no significant differences in total distance traveled or maximum acceleration between F0 mosaic *htr6* crispants and their Cas9 controls. This finding persisted even when animals were retrospectively grouped by the presence or absence of epileptiform discharges. The dissociation between electrophysiological and behavioral measures may stem from several factors. First, the epileptiform discharges were recorded locally from the optic tectum, and such focal activity may be insufficient to activate downstream motor circuits or disrupt global locomotion. Second, the absence of overt behavioral correlates likely reflects the subclinical or interictal-like nature of the discharges, a phenomenon documented in other zebrafish epilepsy models where electrophysiological abnormalities occur independently of detectable behavioral changes^11^, ^19^. Third, the F0 mosaic configuration of our crispants introduces inter-individual variability in editing efficiency and mutation burden, contributing to incomplete penetrance of both electrophysiological and behavioral phenotypes. Collectively, these observations highlight the essential value of integrating electrophysiological recordings with behavioral assessments to construct a comprehensive seizure profile. Relying solely on standard locomotor paradigms risks overlooking subclinical neural hyperexcitability that may lie hidden beneath a behaviorally quiescent surface.

Transcriptomic sequencing might help indicate potential underlying mechanisms. The transcriptome analysis revealed 402 DEGs following *htr6* deficiency, underscoring the functional significance of *htr6* in regulating gene expression related to neuronal development and function. The pronounced up-regulation of genes associated with the mTOR pathway, which plays a pivotal role in cellular physiology and developmental pathways critical for neural health^20^, ^21^. Multiple neurodevelopmental disorders (schizophrenia^22^, epilepsy^3^, etc.) were found to be associated with *HTR6* abnormality, in which mTOR deregulation is increasingly recognized as a key signaling event. This is in accordance with the current research on the transcriptome, as a number of DEGs after *htr6* deficiency were identified in the mTOR signaling pathway. DR enhances memory through the involvement of tryptophan and serotonin receptor HTR6. Inactivation of HTR6 reduces DR-induced neurological changes and implicates *HTR6*-mediated mTORC1 signaling as a nutrient sensor that links memory performance to dietary intake^23^. Previous studies have shown that excessive serotonin signaling via HTR6 affects embryonic interneuron migration, which may contribute to cortical migration defects and excitatory-inhibitory imbalance^24^.

The KEGG enrichment analysis indicates significant involvement of ABC transport pathways. The ABC superfamily of transporters, which could mediate the efflux of compounds out of the CNS, has been increasingly recognized as a key contributor to drug-resistant epilepsy, with FCD receiving particular attention^25^. Notably, *ABCG2* mRNA was found to be significantly upregulated in human FCD tissue samples. This is consistent with the htr6 deficiency model in our study, suggesting that ABC transporters play a critical regulatory role in epileptogenesis^26^. Importantly, our refined GO analysis now reveals that a substantial fraction of DEGs is directly involved in synaptic structure and function. These include genes controlling synaptic vesicle exocytosis, endocytosis, trans-synaptic signaling, and postsynaptic density organization. Dysregulation of these synaptic genes likely impairs neurotransmitter release, receptor trafficking, and synaptic plasticity, all of which are critical for maintaining the excitation-inhibition balance. This molecular derangement provides a direct mechanistic link to the spontaneous epileptiform activity observed in *htr6* deficient larvae. Moreover, the integrated PPI network shows that mTOR, ABC transporter, neural and synaptic proteins are extensively interconnected. Thus, the combined disruption of these pathways plausibly underlies both the morphological brain defects and the hyperexcitability phenotype. The current study further suggests the CNS morphological malformation and electrophysiological dysfunction related to htr6 deficiency except for the up-mentioned circuit dysregulation. This might provide further evidence of *htr6* in the pathogenesis of neurodevelopmental disorders, especially epilepsy. However, further studies are still warranted to determine the precise mechanism within zebrafish *htr6* deficiency.

HTR6 raises particular interest in neurologic disorders as a potential target for treatment. It has been established to regulate cholinergic and glutamatergic neurons and is involved in neurodevelopment, cognition, as well as affective state^5^, ^12^, ^16^. HTR6 interacts with the mechanistic target of mTOR pathway and aberrant signaling has been associated with multiple neurological disorders, including seizure^3^, ^27^-^29^. Interestingly, the mTOR pathway has gained significant attention in research and is considered a novel therapeutic target in the treatment of FCD and epilepsy.

Serotonergic receptors have emerged as one particularly promising target for the treatment of epilepsy^30^. For instance, fenfluramine, a substituted amphetamine derivative that enhances serotonergic transmission by promoting 5-HT release and inhibiting reuptake, has demonstrated substantial efficacy in treatment of Dravet syndrome or other drug-resistant epilepsy^31^, ^32^. It exerts broad modulatory effects on 5-HT₁D, 5 HT₂A, and 5 HT₂C receptors, contributes to stabilization of neuronal excitability and suppression of recurrent seizures^33^, ^34^. In addition, HTR6 antagonists, such as SB-399885^3^, ^35^ and SB-271046^36^, ^37^, have shown anti-epileptic effects in animal model through pathways involving ERK1/2, Fyn^7^ and mTOR^3^. However, clinical data on fenfluramine or HTR6 antagonists used in FCD II patients remains scarce, and future research must rigorously assess potential pharmacodynamic interactions and long-term tolerability. The zebrafish model could serve as a rapid, scalable system for evaluating gene function and acute drug effects on seizure-like events and downstream signaling. At the same time, it does not fully capture the cortical dyslamination, chronic spontaneous seizures, or blood-brain barrier complexity that characterize human FCD II. Future studies using mammalian models that more closely mimic FCD II histopathology will be essential for validating these targets and determining whether serotonergic modulation can be rationally leveraged in this specific patient population. Overall, the investigation of serotonergic modulation, including HTR6-targeted strategies, substantially broadens therapeutic possibilities for drug-resistant epilepsies and provides a rational direction for adjunctive intervention.

Both serotonin antagonists and agonists were found to underlie precognitive function^38^. Preliminary evidence of a beneficial effect modulating serotonin in the maximal electroconvulsive shock test has been established in rat models^37^. In more recent years, the anti-seizure function of HTR6 antagonists was subsequently found to be possibly associated with the activation p-ERK1/2, Fyn^7^ and blockade of mTOR^3^ in animal models with temporal lobe epilepsy (TLE). Brain slices from temporal neocortex after surgical section showed upregulated *HTR6* expression in intractable TLE patients^3^. HTR6 is a favorable druggable target with antagonists already passed phase II clinical trial for cognitive impairment^39^. As a result, drug development might be fast if *HTR6* is validated as an epilepsy-related gene.

Notably, the apparent discrepancy between the anticonvulsant effects of acute HTR6 antagonism and the pro-epileptogenic phenotype of constitutive *htr6* deficiency may be explained by differences in developmental timing. Acute pharmacological blockade acts on already-matured neural networks, in which HTR6 signaling contributes to maintaining excitatory/inhibitory balance^40^. In contrast, constitutive *htr6* deficiency disrupts receptor function during critical developmental windows, when *HTR6* regulates circuit maturation and mTOR signaling, as indicated by our transcriptomic analysis. Consistent with this, *5-HT6R* null mutant mice exhibit increased neuronal excitability and altered neuronal morphology^41^. Furthermore, our *HTR6* mRNA rescue data demonstrate that timely restoration of HTR6 significantly reduces epileptiform discharges, supporting a developmentally dependent mechanism.

The limitations of this study warrant careful consideration. While the utilization of zebrafish as a model organism offers valuable opportunities for in vivo analysis of developmental and behavioral changes, translating these findings to complex human neurological disorders like FCD presents significant challenges. Moreover, the inherent limitations of our approach prevent us from distinguishing whether the observed brain region differences reflect primary malformations or merely delayed neuronal migration, as seen in FCD patients. To address these limitations, future studies will prioritize cross-species validation. In utero electroporation in embryonic mice or human induced pluripotent stem cell-derived organoids will be employed to recapitulate disease-associated cellular phenotypes. A further limitation of this study is the use of F0 mosaic crispants rather than stable germline mutants. While this approach enables rapid functional screening, the inherent mosaicism results in inter-individual variability in editing efficiency and mutation burden, which may contribute to the incomplete penetrance of the epileptiform phenotype. Future studies using stable knockout lines or conditional mutants in mammalian systems will be essential to confirm the causal role of HTR6 in epileptogenesis. Additionally, several technical limitations warrant acknowledgment. The invasive nature of electrophysiological recordings necessitated performing transcriptomic analyses on an independent batch of larvae, thereby precluding paired sampling and stratification by epileptiform status. Sample sizes were determined empirically in the absence of formal power analysis, and the small subgroup sizes in the retrospective behavioral stratification restricted statistical power to detect subtle locomotor differences. These constraints should be taken into account when interpreting the negative behavioral and exploratory transcriptomic findings.

## Conclusion

In conclusion, this study enhances our understanding of the *htr6* gene's role in zebrafish development and its potential implications for neurological disorders, particularly epilepsy. The findings reveal critical insights into the molecular and behavioral consequences of *htr6* deficiency, highlighting its involvement in ocular development, gene expression changes, and seizure predisposition. These results lay the groundwork for future investigations aimed at exploring therapeutic strategies that target HTR6-related pathways, ultimately contributing to the development of more effective interventions for epilepsy and related conditions.

## Supplementary Material

Supplementary methods, figures and tables.

## Figures and Tables

**Figure 1:**
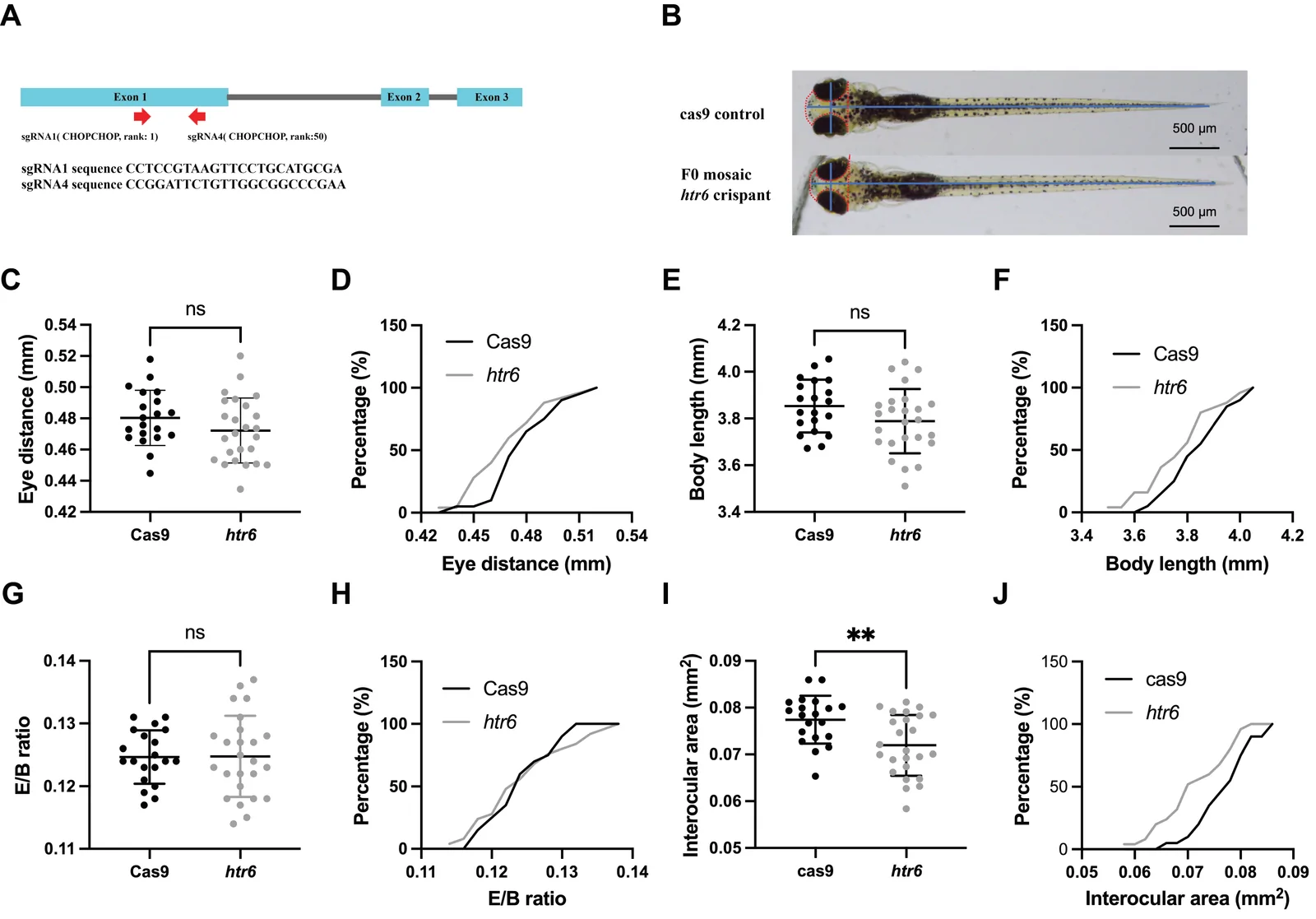
Disruption of zebrafish *htr6* led to abnormal morphology. (A) Schematic diagram of zebrafish *htr6* gene structure and sgRNA targeting strategy. The first exon of zebrafish *htr6* was targeted with two separate CRISPR gRNAs (sgRNA1 and sgRNA4). The positions of the seven transmembrane helices and the putative ligand-binding domain are indicated relative to the open reading frame. sgRNA1 is located between TM helices 4 and 5, and sgRNA4 is positioned near TM helices 5 and 6. (B) Representative bright-field imaging of larval zebrafish at 5 dpf (dorsal view). Top, Cas9 control; bottom, F0 mosaic *htr6* crispant. (C-D) Measurements and histogram of eye distance in Cas9 control (n = 20) vs. F0 mosaic *htr6* crispant (n = 25). (E-J) Measurements and histogram of body length (E-F), E/B ratio (G-H) and interocular area (I-J) in Cas9 control (n = 20) vs. F0 mosaic* htr6* crispant (n = 25). Scale bars as indicated in the figure. Bars represent mean ± SD; unpaired *t*-test. Statistical significance is indicated as **p < 0.01.

**Figure 2:**
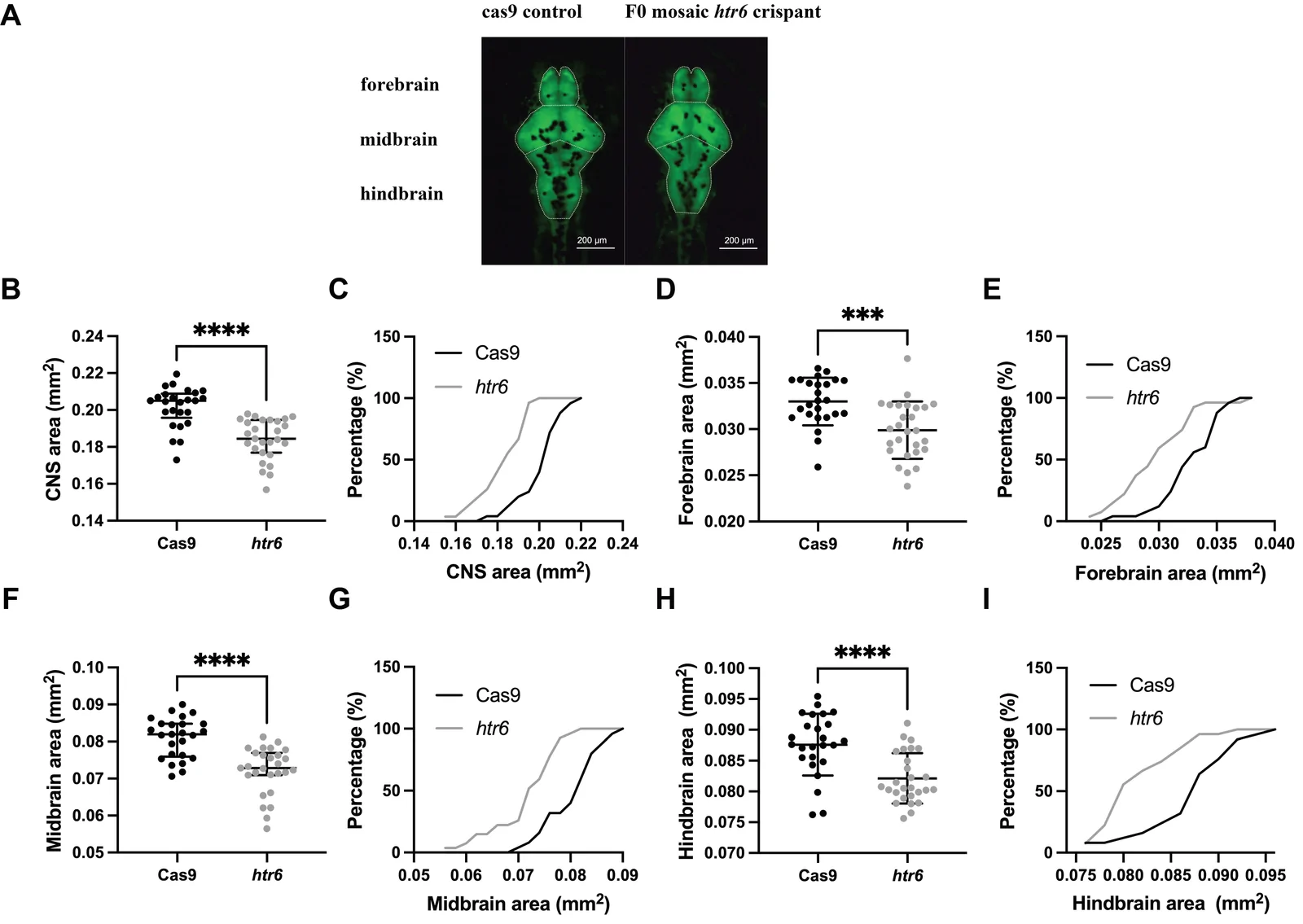
Disruption of zebrafish *htr6* led to abnormal central nervous system (CNS) morphology. Data from the initial characterization cohort. (A) Representative imaging of HuC: eGFP expressed larval zebrafish shows CNS fluorescence pattern at 5 dpf (dorsal view). Left, Cas9 control; right, F0 mosaic *htr6* crispant. White dash line highlights the CNS subregions, including the forebrain, midbrain, and hindbrain, for measurement. Scale bars as indicated in the figure. (B-C) Measurements and histogram of CNS area in Cas9 control (n=25) vs. F0 mosaic *htr6* crispant (n=27). Bars represent median and IQR; Mann-Whitney U test. (D-I) Measurements and histogram of CNS subregion area, including forebrain area (D-E, bars represent mean±SD; unpaired *t*-test), midbrain area (F-G, bars represent median and IQR; Mann-Whitney U test) and hindbrain area (H-I, bars represent mean±SD; unpaired *t*-test) in Cas9 control (n=25) vs. F0 mosaic *htr6* crispant (n=27). Statistical significance is indicated as ***p < 0.001 and ****p < 0.0001.

**Figure 3:**
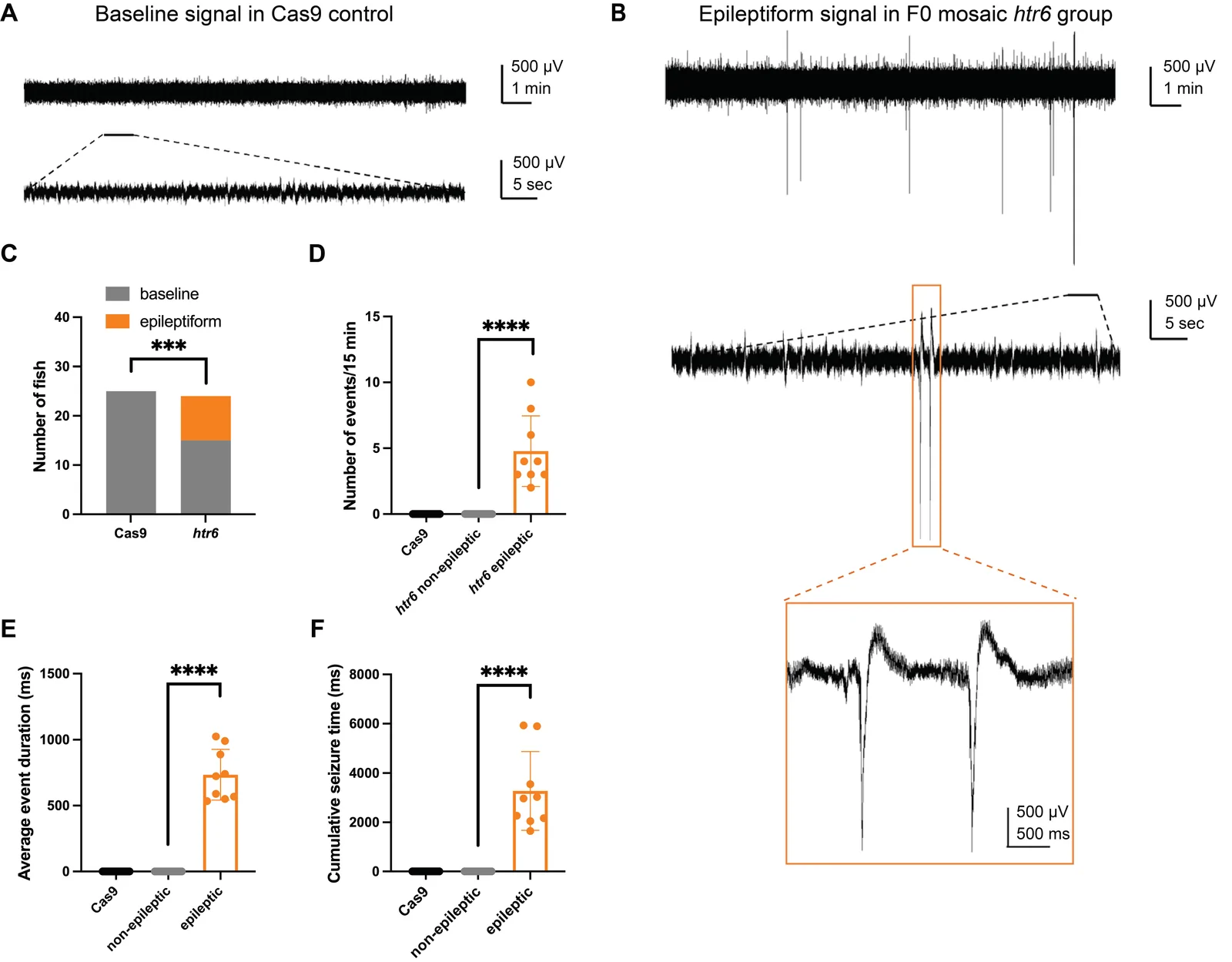
Impact of *htr6* deficiency in zebrafish larvae on electrophysiology. Data from the initial characterization experiment. (A) Representative electrophysiology record observed in Cas9 control group. (B) Representative epileptiform signal observed in F0 mosaic *htr6* crispant group. (C) Statistical analysis of zebrafish with epileptiform signal between Cas9 control group and F0 mosaic *htr6* crispant group. Fisher's exact test. (D) Statistical analysis of the number of events per 15 minutes recording period. (E) Statistical analysis of the average duration of individual epileptiform events. (F) Statistical analysis of the cumulative seizure time per fish. (D-F) Bars represent mean ± SD; unpaired *t*-test. Statistical significance is indicated as ***p < 0.001 and **** *p* <0.0001.

**Figure 4:**
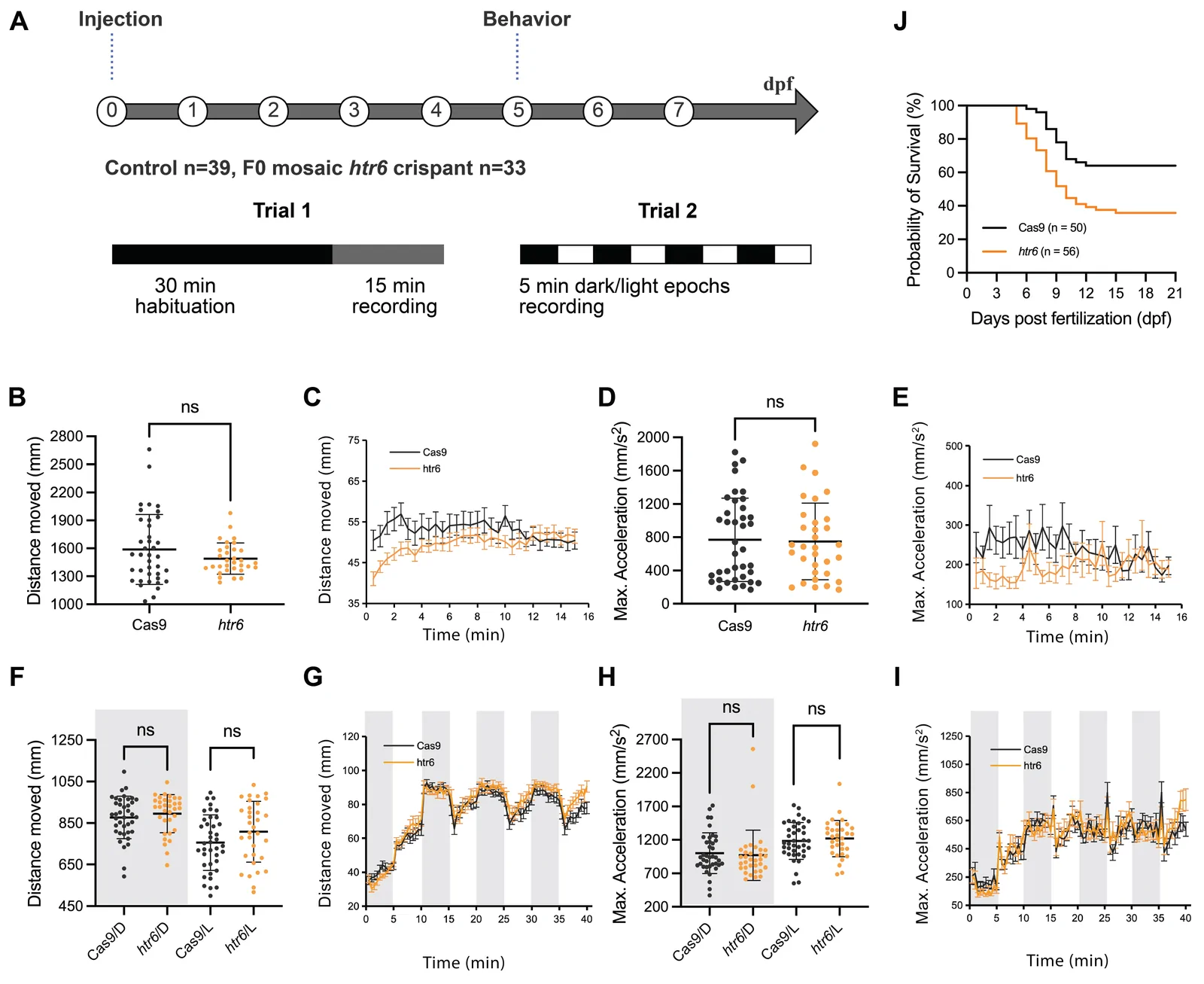
Disruption of zebrafish *htr6* has no effect on locomotor behavior but reduces survival. (A) Design of Behavioral Locomotor Assays. Trial 1: 15-minutes tracks of spontaneous behavior following 30 minutes of habituation in the dark. Trial 2: Locomotor behavior at 5-min dark/light epochs, totaling 4 epochs. (B-E) Statistical analysis and line chart and of the distance moved and the maximum acceleration data of the Cas9 control group and the F0 mosaic *htr6* crispant group in trial 1. (F-I) Line charts and statistical analysis of the distance moved and the maximum acceleration data of the Cas9 control group and the F0 mosaic *htr6* crispant group in trial 2. In the figure, white areas denote measurement data collected in a bright environment, whereas gray areas represent data from a dark environment. (B-I) Scale bars as indicated in the figure. Bars represent mean ± SD; unpaired *t*-test. (J) Kaplan-Meier survival curve of Cas9 control and F0 mosaic *htr6* crispant groups. Log-rank (Mantel-Cox) test: χ² = 10.25, df = 1, *p* = 0.001. Cas9 control n = 50, F0 mosaic *htr6* crispant n = 56.

**Figure 5:**
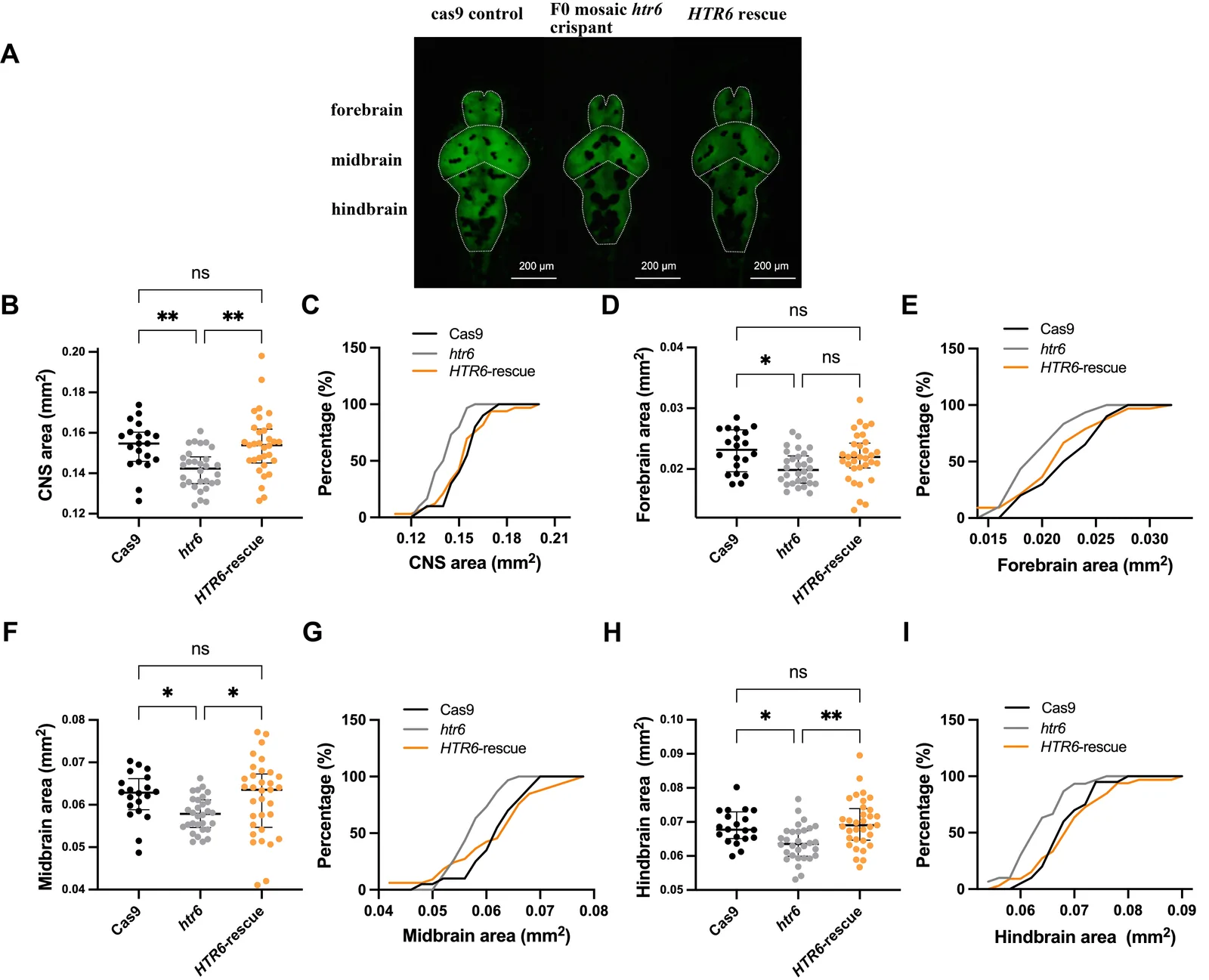
Human *HTR6* rescues the CNS developmental abnormalities in F0 mosaic *htr6* crispant group. Data from the rescue experiment cohort. (A) Representative imaging of HuC: eGFP expressed larval zebrafish shows CNS fluorescence pattern at 5 dpf (dorsal view). Left, Cas9 control; middle, F0 mosaic *htr6* crispant; right, F0 mosaic *htr6* crispant +*HTR6* mRNA. White dash line highlights the CNS subregions, including forebrain, midbrain, and hindbrain, for measurement. Scale bars as indicated in the figure. (B-C) Measurements and histogram of CNS area in Cas9 control (n = 20), F0 mosaic *htr6* crispant (n = 30) and F0 mosaic *htr6* crispant +*HTR6* mRNA (n = 33). (D-I) Measurements and histogram of CNS subregion area, including forebrain area (D-E), midbrain area (F-G), and hindbrain area (H-I) in Cas9 control (n=20), F0 mosaic *htr6* crispant (n=30), and F0 mosaic *htr6* crispant +*HTR6* mRNA (n=33). Bars represent median and IQR; Kruskal-Wallis test, followed by Dunn's multiple comparisons test for post hoc analysis. Statistical significance is indicated as *p < 0.05 and **p < 0.01.

**Figure 6:**
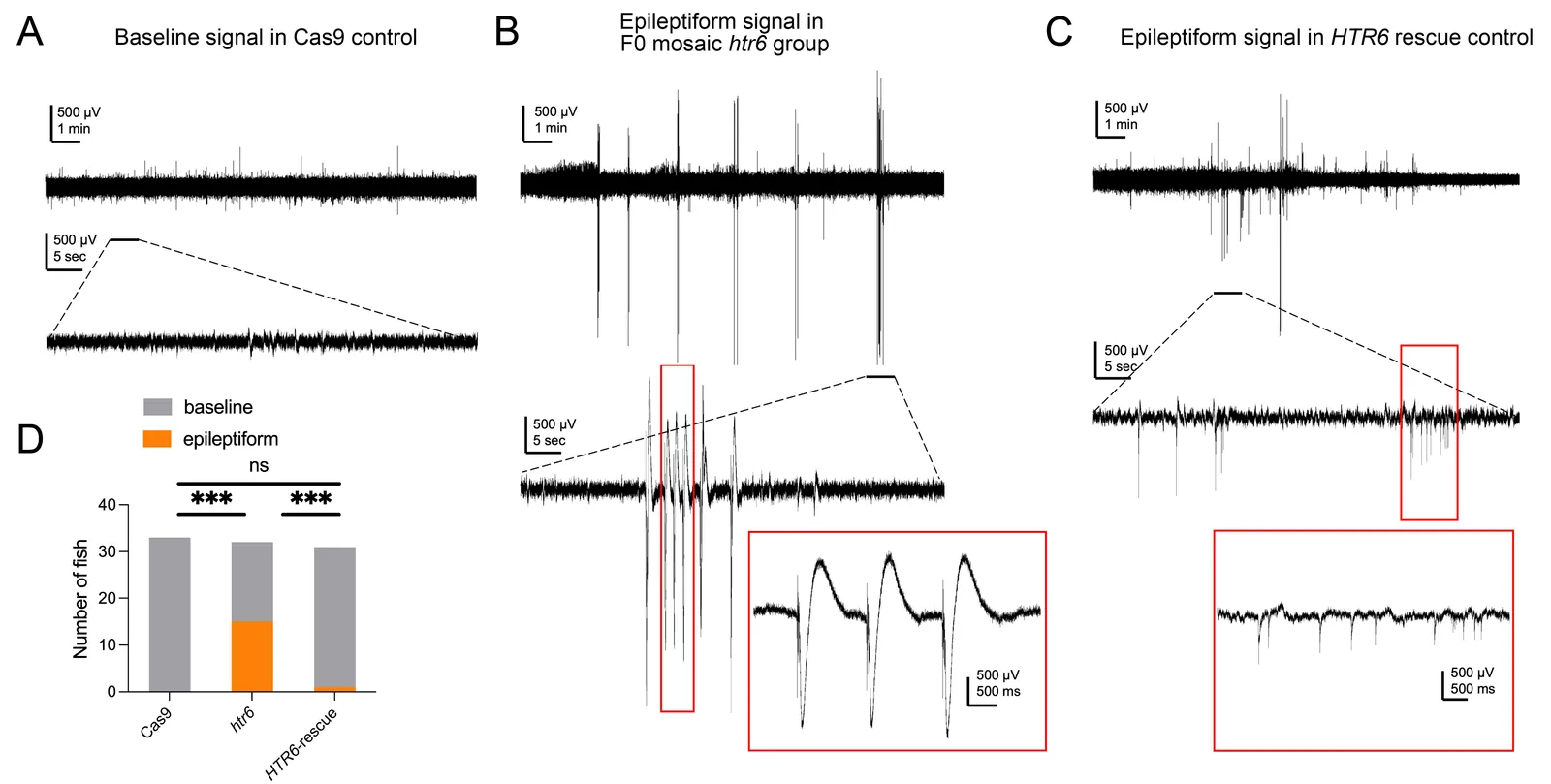
Human *HTR6* rescues the epileptiform activity in F0 mosaic *htr6* crispant group. Data from the rescue experiment cohort. (A) Representative electrophysiology record observed in Cas9 control group. (B) Representative epileptiform signal observed in F0 mosaic *htr6* crispant group. (C) Representative epileptiform signal observed in F0 mosaic *htr6* crispant +*HTR6* mRNA group. (D) Statistical analysis of zebrafish with epileptiform signal between Cas9 control group, F0 mosaic *htr6* crispant group and F0 mosaic *htr6* crispant +*HTR6* mRNA group. Fisher's exact test; statistical significance is indicated as *** *p* < 0.001.

**Figure 7:**
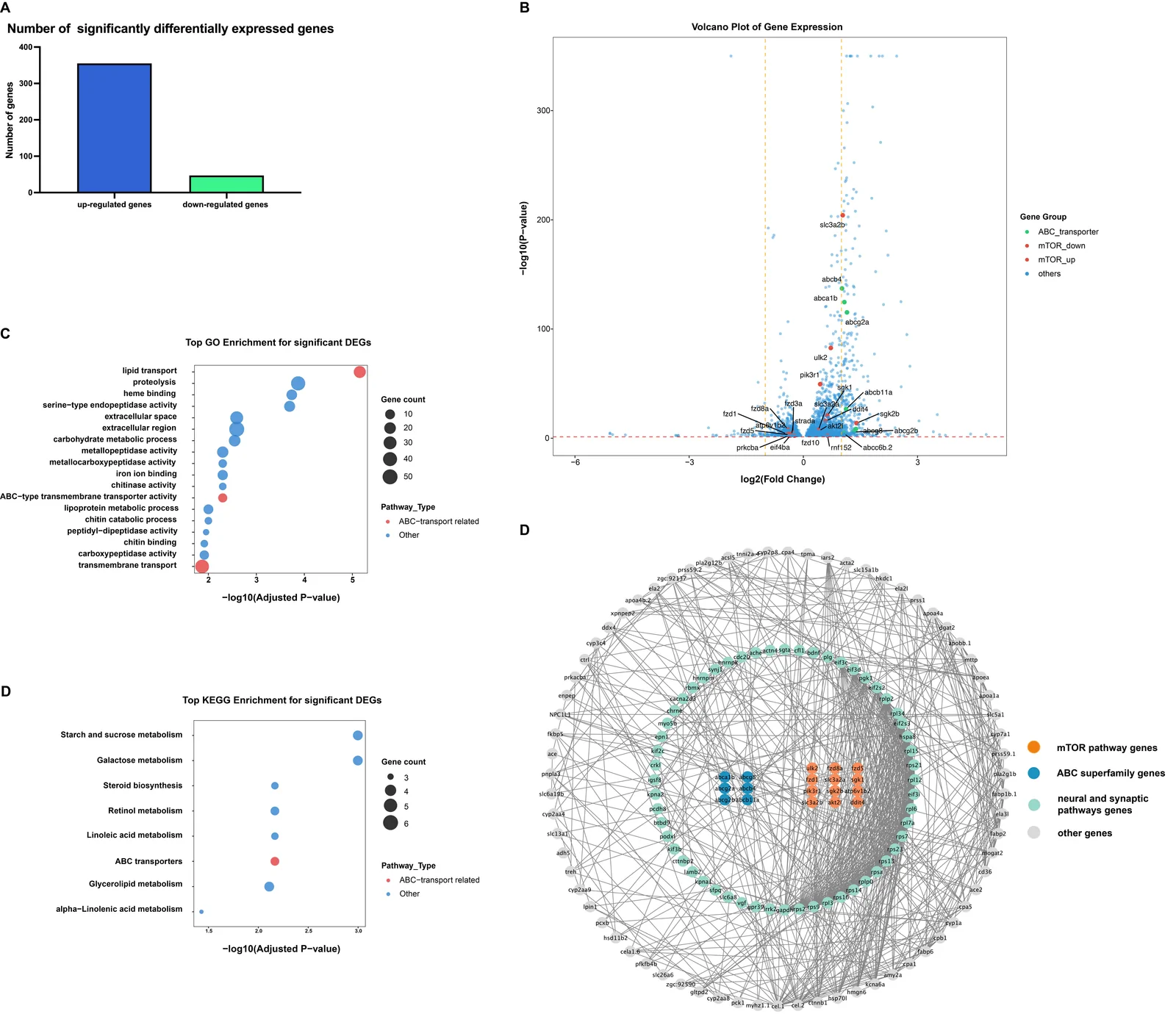
Transcriptome sequencing revealed differentially expressed genes and top pathways associated with *htr6* deficiency. (A) Number of significantly differentially expressed genes. (B) Volcano plot of the differentially expressed genes. Green points, differentially expressed genes enriched from the mTOR pathway; red points, ABC superfamily genes. (C) dot plot for GO enrichment of all significant differentially expressed genes. (D) dot plot for KEGG enrichment of all significant differentially expressed genes. (E) Protein-protein interaction analysis of significant differentially expressed mTOR pathway genes and ABC superfamily genes.

**Figure 8:**
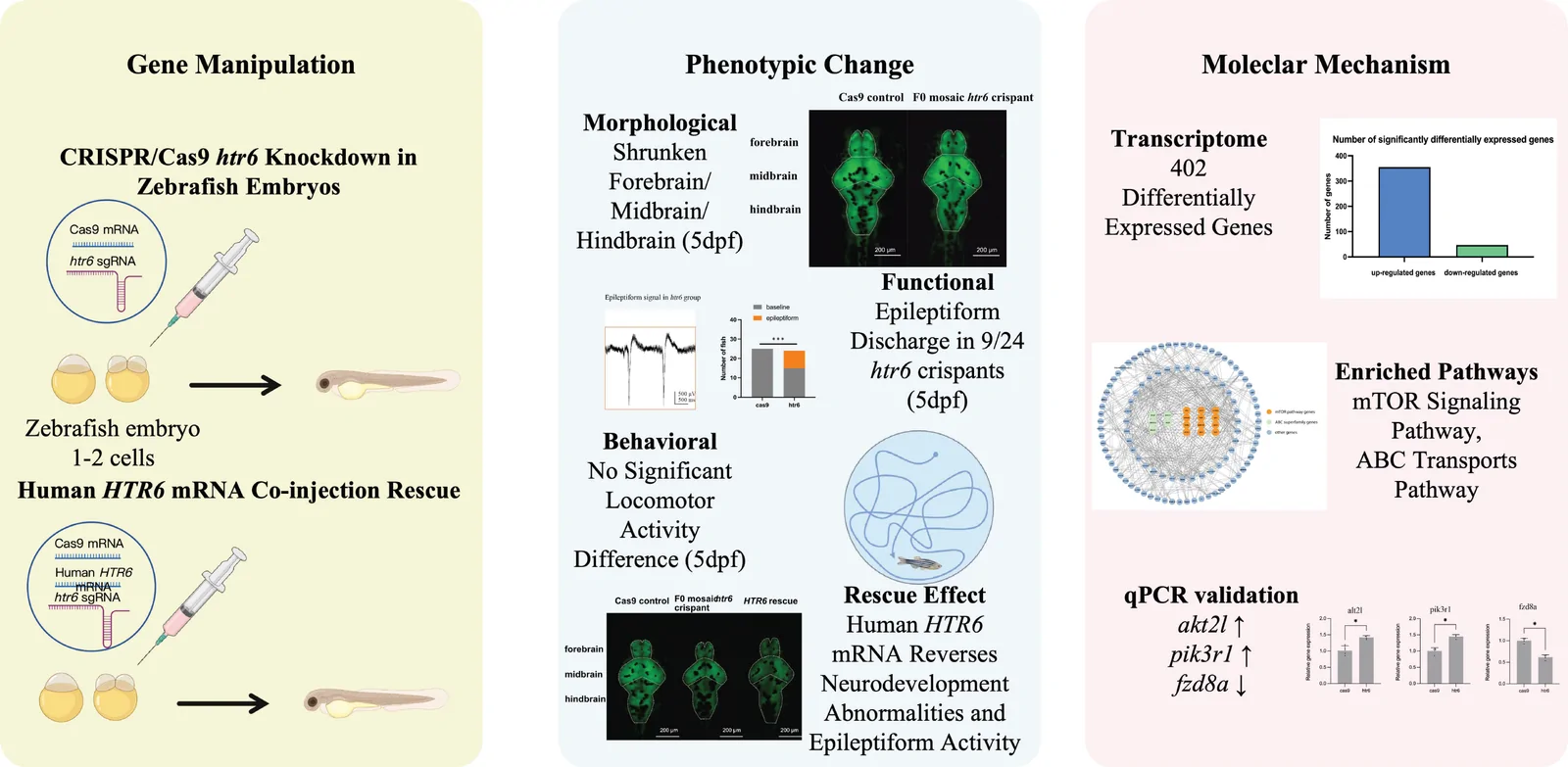
Graphical abstract, CRISPR/Cas9-mediated *htr6* deficiency in zebrafish reveals neurodevelopmental defects and epileptiform activity. Deficiency of *htr6* in zebrafish severely impairs central nervous system development, triggering diminished brain region size and intense epileptiform neural hyperactivity. Strikingly, human *HTR6* mRNA substantially rescues brain morphology and potently suppresses epileptiform discharges.

## Data Availability

The datasets generated during and/or analyzed during the current study are available from the corresponding author on reasonable request after review and approval of the Institutional Review Board.

## Abbreviations

5-HT: 5-hydroxytryptamine
ABC: ATP-binding cassette
CNS: central nervous system
DEGs: differentially expressed genes
dpf: days post-fertilization
DR: dietary restriction
FCD: focal cortical dysplasia
FPKM: fragments per kilobase of transcript per million mapped reads
GO: Gene Ontology
HTR6: 5-hydroxytryptamine receptor 6
IQR: interquartile range
KEGG: Kyoto Encyclopedia of Genes and Genomes
mTOR: mechanistic target of rapamycin
PCR: polymerase chain reaction
PPI: protein-protein interaction
SD: standard deviation
TIDE: tracking of indels by decomposition
TLE: temporal lobe epilepsy
TM: transmembrane
