# Supplementary material for: The deficiency of htr6 gene results in aberrant brain development and epileptiform discharges in zebrafish larvae
Source: Int J Med Sci. 2026 Oct 2;23(11):3515–30. doi: 10.7150/ijms.135746 (PMC13685442; doi:10.7150/ijms.135746)

Human–zebrafish HTR6 conservation

A pairwise alignment between human HTR6 and zebrafish htr6 was generated using DIOPT v10 (https://www.flyrnai.org/cgi-bin/DRSC_prot_align.pl). The alignment spans 470 residues, featuring 239 identical residues (50%), 276 similar residues (58%), and 94 gaps (20%). Within the class–A GPCR seven–transmembrane domain (CDD: 7tmA), 203 out of 354 residues are identical (57%). Per–helix conservation ranges from moderate to high: TM1 at 46% (12/26), TM2 at 88% (22/25), TM3 at 83% (24/29), TM4 at 68% (15/22), TM5 at 78% (18/23), TM6 at 69% (20/29), and TM7 at 79% (19/24). Canonical motifs are preserved, including SLDRY (end of TM3) and NPxxY (TM7), and the extracellular cysteines supporting the conserved disulfide bond. Conversely, extramembrane regions, particularly the disordered C–terminal tail, show markedly lower conservation (7/45, 16%). These findings underscore robust conservation of the ligand–binding/transmembrane core, affirming zebrafish as a translationally relevant model for probing essential HTR6 functions.

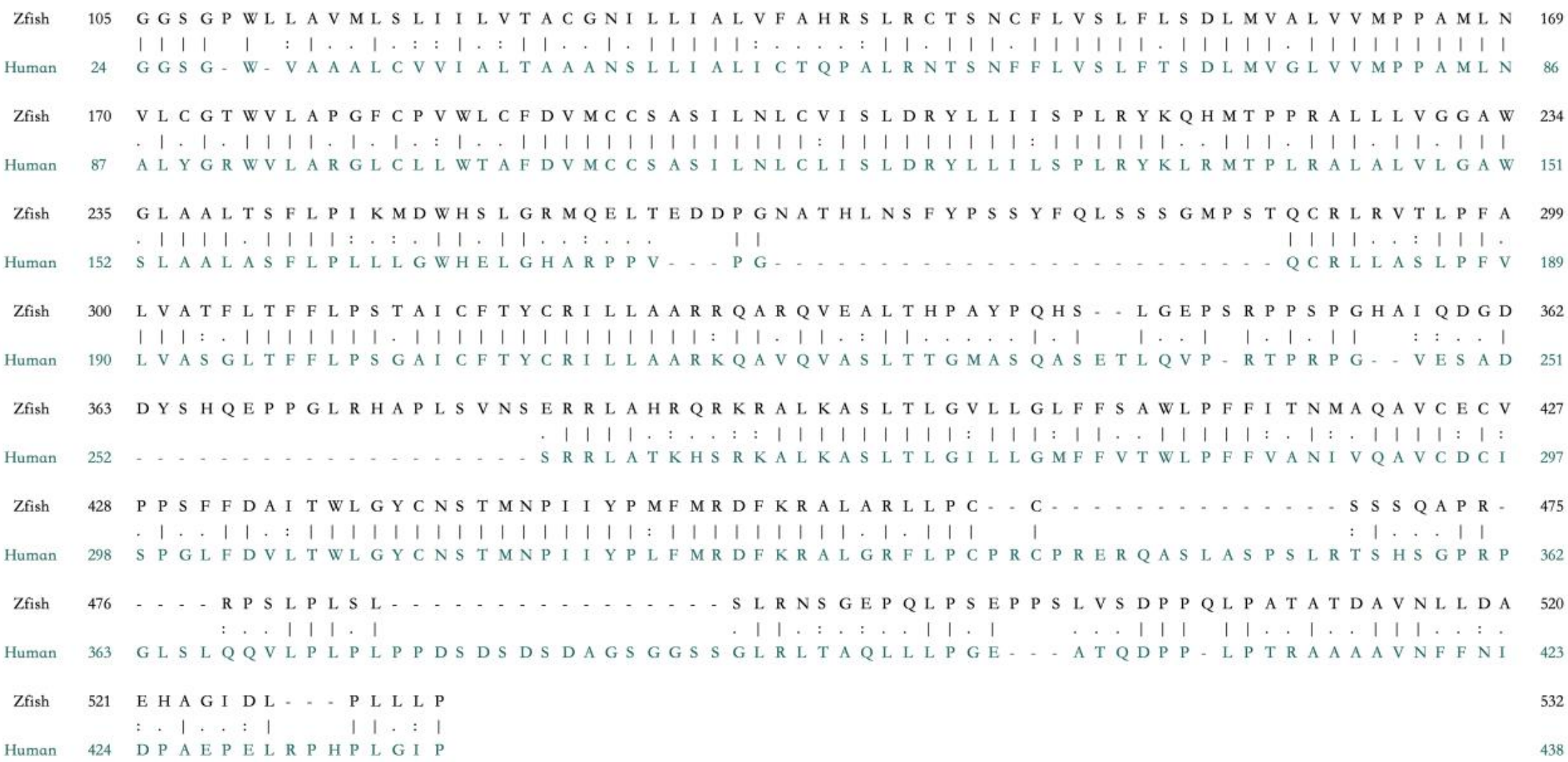

Figure S1. Protein alignment of human HTR6 (NP_000862.1) and zebrafish htr6 (XP_009295353.2). Vertical bars indicate identical residues, colons conservative substitutions, periods semi-conservative substitutions; dashes denote gaps. Across the aligned region (470 aa), overall identity is 50%, similarity 58% and gaps 20%.

Table S1. Per-domain and per-helix sequence identity between human HTR6 (NP_000862.1) and zebrafish htr6 (XP_009295353.2).

	Sequence	Domain	Region	External ID	Identity
**Zebrafish htr6**	XP_009295353.2	None			
**Human HTR6**	NP_000862.1	7tmA_5–HT6	27..331	CDD:320182	203/354 (57%)
		TM helix 1	28..54	CDD:320182	12/26 (46%)
		TM helix 2	61..87	CDD:320182	22/25 (88%)
		TM helix 3	99..129	CDD:320182	24/29 (83%)
		TM helix 4	141..164	CDD:320182	16/22 (73%)
		TM helix 5	185..214	CDD:320182	23/28 (82%)
		TM helix 6	261..291	CDD:320182	20/29 (69%)
		TM helix 7	299..324	CDD:320182	19/24 (79%)
		disordered C–terminal tail	346..392		7/45 (16%)

Table S2. Gene editing efficiency of F0 mosaic *htr6* crispants

**htr6 knock–down ID**	**efficiency**	**htr6 knock–down ID**	**efficiency**	**htr6 knock–down ID**	**efficiency**
E2	96%	G6	Sequence failure	9	79%
E3	100%	G7	22%	10	73%
E4	99%	G8	91%	11	100%
E5	Sequence failure	G9	29%	12	100%
E6	36%	G10	12%	13	100%
E7	37%	G11	100%	14	75%
E8	26%	H2	56%	15	100%
E9	94%	H3	98%	16	65%
E10	100%	H4	90%	17	100%
E11	100%	H5	97%	18	100%
F2	96%	H6	Sequence failure	19	100%
F3	70%	H7	78%	20	67%
F4	3%	H8	100%	21	100%
F5	79%	H9	10%	22	100%
F6	100%	H10	0%	23	100%
F7	35%	H11	30%	24	100%
F8	95%	1	82%	25	100%
F9	77%	2	100%	26	100%
F10	96%	3	99%	27	92%
F11	100%	4	98%	28	100%
G2	1%	5	100%	29	100%
G3	100%	6	65%	30	100%
G4	Sequence failure	7	100%		
G5	14%	8	100%		

Table S3. Gene editing efficiency of *HTR6*–rescue zebrafish larvae

HTR6–rescue	efficiency	*HTR6*–rescue	efficiency	*HTR6*–rescue	efficiency
1	100%	15	0%	29	83%
2	100%	16	83%	30	99%
3	0%	17	0%	31	100%
4	98%	18	0%	32	100%
5	0%	19	0%	33	100%
6	96%	20	0%	34	100%
7	90%	21	24%	35	100%
8	100%	22	96%	36	100%
9	99%	23	85%	37	100%
10	100%	24	100%	38	100%
11	100%	25	100%	39	79%
12	43%	26	100%	40	100%
13	0%	27	100%	41	100%
14	0%	28	99%	42	99%

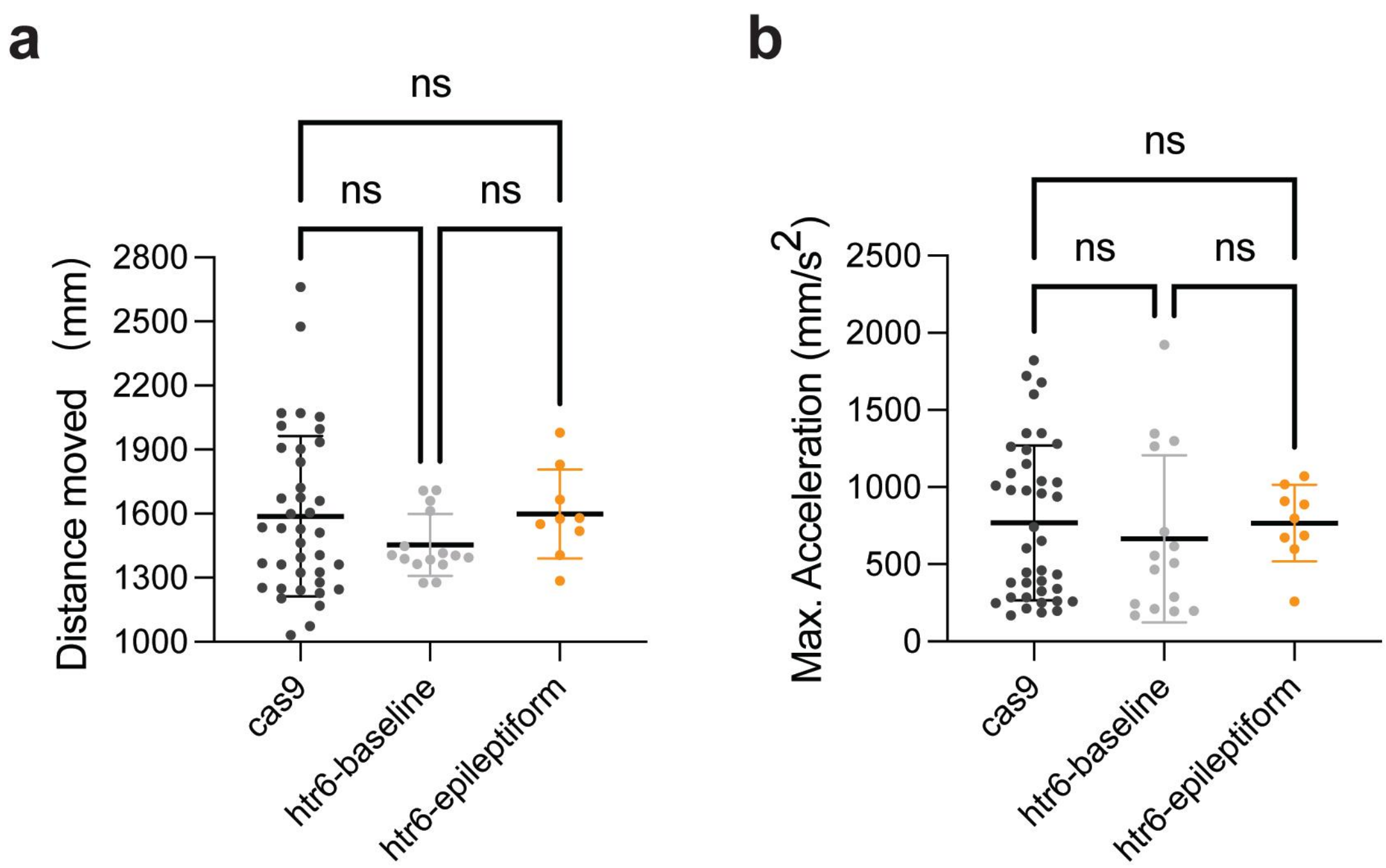

Figure S2. Spontaneous locomotor activity is not altered in F0 mosaic *htr6* crispant with epileptiform signals. Total distance moved (a) and maximum acceleration (b) were comparable among Cas9 controls, F0 mosaic *htr6* crispant without epileptiform signals (*htr6*–baseline), and F0 mosaic *htr6* crispant with epileptiform signals (*htr6*–epileptiform). Data are shown as mean ± SD; Tukey's multiple-comparisons test.

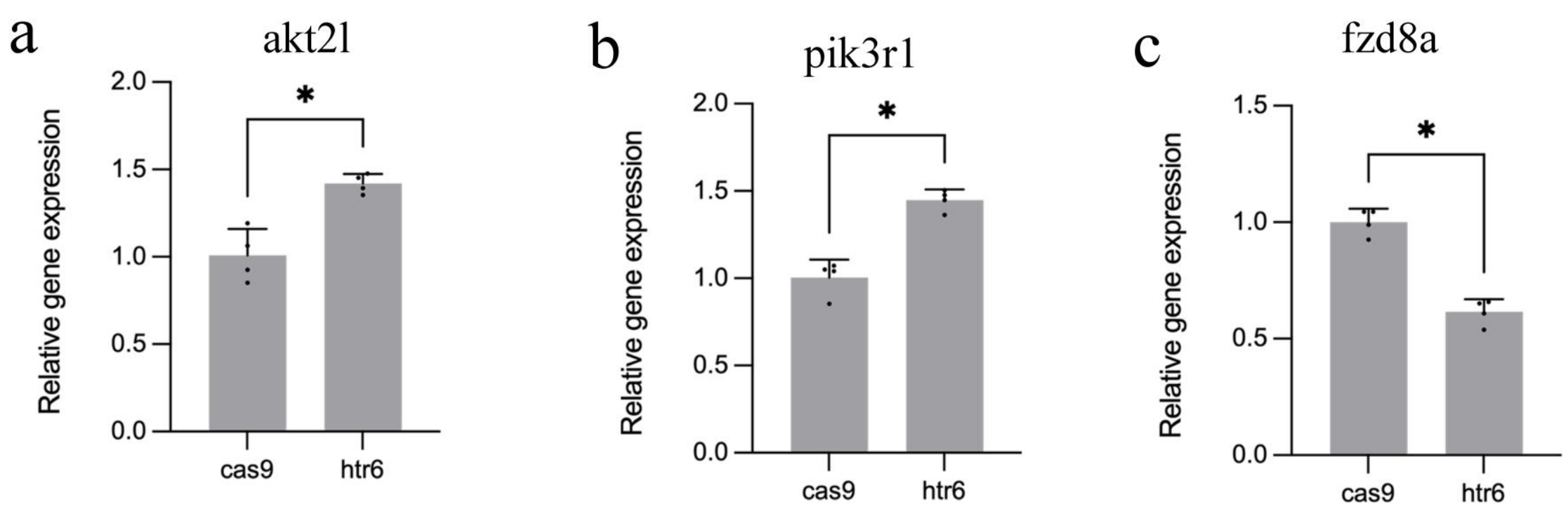

Figure S3. Validation of mTOR-related gene expression changes in htr6 crispants by qPCR. qPCR analysis showing upregulation of akt2l (a) and pik3r1 (b), downregulation of fzd8a (c) in htr6 crispants compared with Cas9 controls. Data are presented as meanmean±quartile; Mann–Whitney test.

Table S4. Primer sequences utilized for qPCR

Gene	Forward primer (F)	Reverse Primer (R)	Product Size (bp)
akt2l	GCGCTCGTAGTAAACAGGAGT	CGGCCTCCATGTCTTGATGT	131
pik3r1	GATCGGACTTTGGTCCGTGT	CAAACATGTAGCACGCCGAG	159
fzd8a	ACCCAGGAAAAGGGGGACTT	CAGAGTGCTCAAGCTTTGCT	128
actb	ACAGAGAGAAGATGACACAGATCATG	AGTCCATCACAATACCAGTAGTACG	120

Table S5. Equipment and reagent utilized in the current study

**Equipment/reagent**	**Model**	**Brand**
Zebrafish Feeding System	Z-A-D5	Hai-Sheng
Stereomicroscope	SMZ800N	Nikon
Dry Block Thermostat	BG-32	Lang-Ji
Glass Electrode Puller	P-97	Sutter
Zebrafish Behavior Tracking System	DVOC-0041	Noldus
Pure Water System	Smart-S15	He-Tai
Biochemical Incubator	LRH-150	Yi-Heng
Electrophysiological Signal Amplifier	1700	A-M Systems
Analog-to-Digital Converter	USB-231	Measurement Computing
Methylene Blue 2.0% Solution	YHSZ-003	Ya-Hua
Low Melting Agarose	A600015-0025	Sheng-Gong
Sodium Chloride	10019318	Hu-Shi
Pancuronium Bromide	P1918	Sigma
RNA–easy Isolation Reagent	No. R701-01	Vazyme
HiScript III 1st Strand cDNA Synthesis Kit	No. R312-01	Vazyme
AceQ qPCR SYBR Green Master Mix	No. R111-02	Vazyme

Table S6. Sample size accounting for each experimental assay, including excluded samples and final n per group.

<table>
<tr><th>Experimental assay</th><th>Excluded due to sequencing failure</th><th>Excluded due to editing efficiency <5%</th><th>Excluded due to recording/ tracking loss</th><th>Final n (Cas 9)</th><th>Final n (F0 mosaic htr6 crispant)</th><th>Final n (HTR6 –rescue)</th></tr>
<tr><td>Morphology</td><td>3</td><td>2</td><td>–</td><td>20</td><td>25</td><td>–</td></tr>
<tr><td>CNS area</td><td>–</td><td>5</td><td>–</td><td>25</td><td>27</td><td>–</td></tr>
<tr><td>Electrophysiology</td><td>3</td><td>2</td><td>1</td><td>25</td><td>24</td><td>–</td></tr>
<tr><td>Locomotor behavior</td><td>4</td><td>3</td><td>1/0*</td><td>39</td><td>32/33*</td><td>–</td></tr>
<tr><td>Rescue morphology (F0 mosaic htr6 crispant)</td><td>–</td><td>–</td><td>–</td><td>20</td><td rowspan="2">30</td><td>–</td></tr>
<tr><td>Rescue morphology (HTR6–rescue)</td><td>–</td><td>9</td><td>–</td><td></td><td>33</td></tr>
<tr><td>Rescue electrophysiology (F0 mosaic htr6 crispant)</td><td>3</td><td>2</td><td>1</td><td>33</td><td rowspan="2">32</td><td></td></tr>
<tr><td>Rescue electrophysiology (HTR6–rescue)</td><td>–</td><td>9</td><td>–</td><td></td><td>31</td></tr>
</table>

* Spontaneous phase n=32 (one excluded due to tracking loss); dark/light epoch phase n=33.

– Indicates not applicable.

Figure S4. Sequence alignment of htr6 exon 1 PCR product against reference genomic sequence

sgRNA1

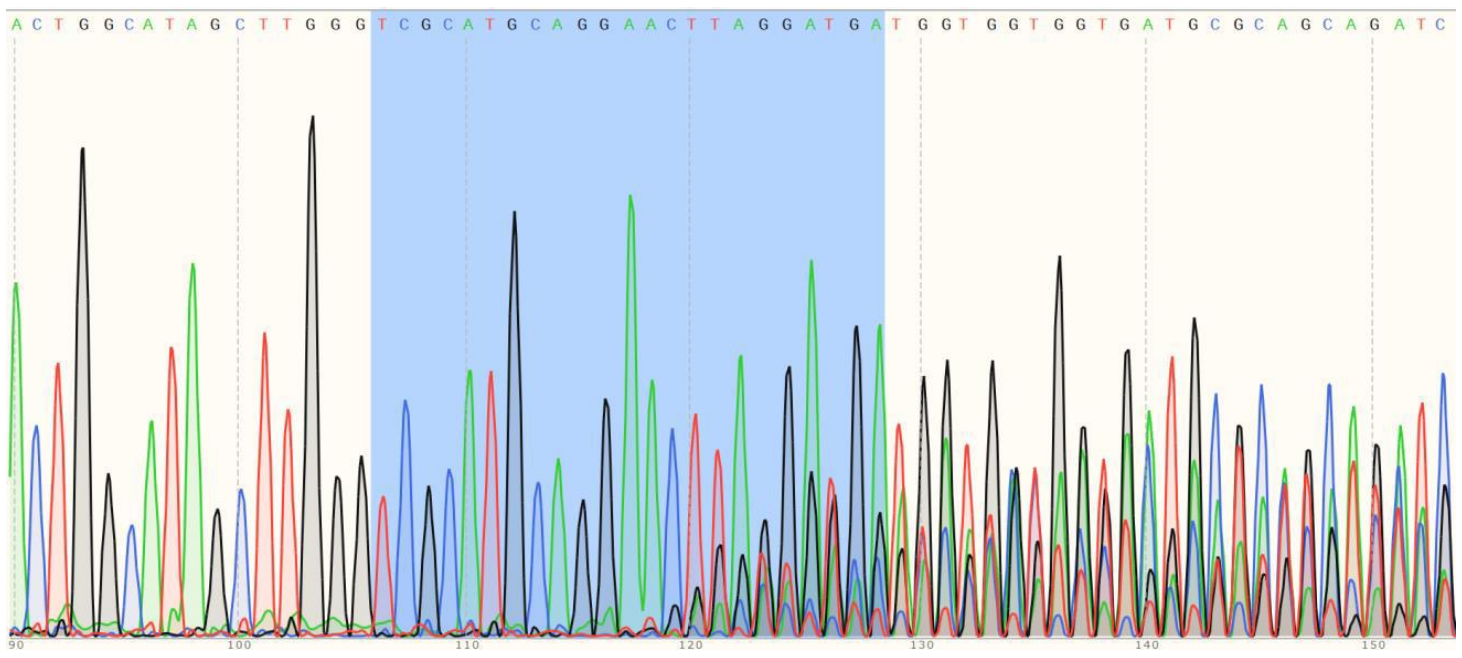

sgRNA4

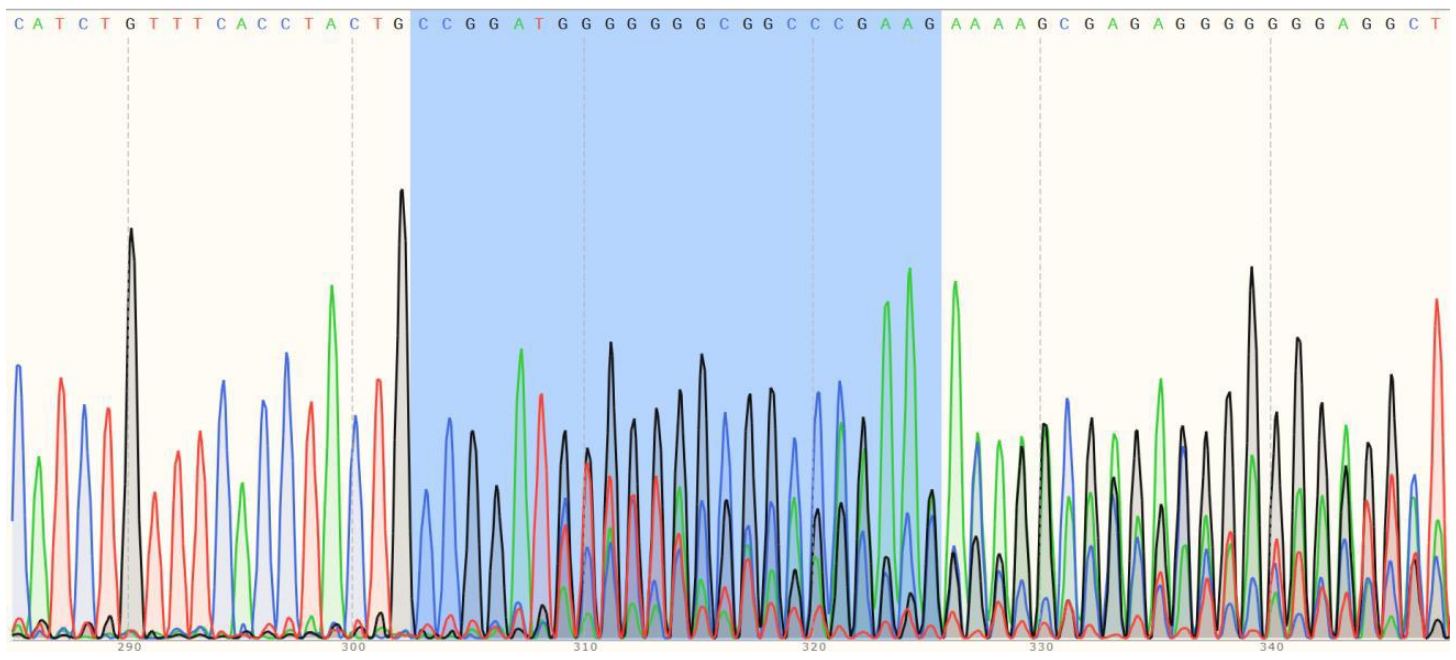

Supplement: Supplementary file 1 [file ijmsv23p3515s1.pdf]
